# Singularities of Pyogenic Streptococcal Biofilms – From Formation to Health Implication

**DOI:** 10.3389/fmicb.2020.584947

**Published:** 2020-12-23

**Authors:** Cinthia Alves-Barroco, João Paquete-Ferreira, Teresa Santos-Silva, Alexandra R. Fernandes

**Affiliations:** ^1^UCIBIO, Departamento Ciências da Vida, Faculdade de Ciências e Tecnologia, Universidade Nova de Lisboa, Campus de Caparica, Caparica, Portugal; ^2^UCIBIO, Departamento de Química, Faculdade de Ciências e Tecnologia, Universidade Nova de Lisboa, Campus de Caparica, Caparica, Portugal

**Keywords:** biofilm, antibiotic resistance, virulence factors, quorum-sensing, streptococcal

## Abstract

Biofilms are generally defined as communities of cells involved in a self-produced extracellular matrix adhered to a surface. In biofilms, the bacteria are less sensitive to host defense mechanisms and antimicrobial agents, due to multiple strategies, that involve modulation of gene expression, controlled metabolic rate, intercellular communication, composition, and 3D architecture of the extracellular matrix. These factors play a key role in streptococci pathogenesis, contributing to therapy failure and promoting persistent infections. The species of the pyogenic group together with *Streptococcus pneumoniae* are the major pathogens belonging the genus *Streptococcus*, and its biofilm growth has been investigated, but insights in the genetic origin of biofilm formation are limited. This review summarizes pyogenic streptococci biofilms with details on constitution, formation, and virulence factors associated with formation.

## Introduction

In nature, bacteria can exist in the planktonic form, where cells live freely in solution, but the sessile form, where a community grows enclosed in an extracellular polymer matrix while attached to a biotic or abiotic surface, is far more common (Donlan, 2002). This phenotype corresponds to a biofilm and can occur on virtually all types of surfaces (Garrett et al., 2008; Flemming and Wingender, 2010; Jamal et al., 2018). The biofilm consists of a tri-dimensional complex community of genetically similar or distinct cells that produce a matrix of extracellular polymeric substances (EPS), which accounts for 80% of the structures. The EPS contains a mixture of alginates, extracellular teichoic acid (TA), proteins, poly-N-acetyl glucosamine, lipids, phospholipids, polysaccharides, and extracellular DNA (e-DNA). About 97% of the EPS is composed of water, which is found as a solvent, dictating viscosity and mobility (Sutherland, 2001; Lu and Collins, 2007; Flemming and Wingender, 2010; Kumar et al., 2017; Jamal et al., 2018).

Biofilm formation is part of a defense mechanism that bacteria adopt to achieve a favorable environment, keep nutrients and increase the chances of survival (Chen and Wen, 2011; Young et al., 2016; Del Pozo, 2018; Jamal et al., 2018; Khatoon et al., 2018).

It is known that biofilms play an important role in streptococci pathogenesis, namely species that belong to the microbiota flora of animals and humans, but also species that are restricted human pathogens. These can cause opportunistic infections, that under appropriate conditions give rise to localized and systemic infections, with considerable implications on public health and veterinary industry (Pires et al., 2005; Rato et al., 2013; Barnett et al., 2015; Peters, 2017). Streptococci are divided into groups based on 16S rRNA gene sequence analysis: “Pyogenic,” “Sanguis,” “Bovis,” “Mutans,” “Mitis,” “Anginosus,” and “Salivarius” (Lal et al., 2011). Afterward, the “Downei” group was created to accommodate *Streptococcus downei* and *Streptococcus criceti*. However, some relationships between groups are not fully understood. This probably reflects the effect of horizontal gene transfer (HGT) during the early diversification of these clusters (Richards et al., 2014). In Table 1, a description of S*treptococcus* species with clinical and veterinary relevance is presented. The pyogenic Streptococci as *Streptococcus pyogenes, Streptococcus agalactiae, Streptococcus dysgalactiae* subsp. *dysgalactiae* (SDSD), and *Streptococcus dysgalactiae* subsp. *equisimilis* (SDSE), together with *Streptococcus pneumoniae* are the major pathogens belonging the genus *Streptococcus* (Parks et al., 2015).

**TABLE 1:** S*treptococcus* species of clinical and veterinary importance.

Group	Species	Lancefield group	Main host	Clinical manifestations	References
Anginosus	*S. anginosus*	A, C, F, G	Human	Bacteremia	Giuliano et al., 2012; Noguchi et al., 2015
	*S. intermedius*	A, C, F, G	Dogs	Bacteremia and abscesses with active periodontal	Tran et al., 2008
Bovis	*S. bovis*	D	Bovine	Endocarditis, bacteremia, meningitis, septicemia, inflammatory gastrointestinal	Dekker, 2016
Mitis	*S. pneumonia*	Viridans	Human	Acute conjunctivitis, meningitis, otitis media, pleural empyema, pneumonia, septic arthritis, septicemia	Henriques-Normark and Tuomanen, 2013
	*S. mitis*	Viridans	Human	Throat infection, bacteremia, endocarditis	Shelburne et al., 2014; Basaranoglu et al., 2019
	*S. oralis*	Viridans	Human		
Mutans	*S. mutans*	Viridans	Human	Dental caries, bacteremia, endocarditis, septicemia	Kojima et al., 2012
	*S. sabrinus*	Viridans	Human		Berlutti et al., 2010
Pyogenic	*S. pyogenes*	A	Human	Pharyngitis, septicemia, necrotizing fasciitis, bacteremia, meningitis, pneumonia, septic arthritis, rheumatic streptococcal toxic shock syndrome, scarlet fever	Carapetis et al., 2005; Cole et al., 2011; Reglinski and Sriskandan, 2014; Isaacs and Dobson, 2016
	*S. agalactiae*	B	Human and Bovine	Human: cellulites, septicemia, pneumonia, meningitis, tract infection, puerperal sepsis, endometirosis, cystitis, bacteremia, Bovine mastitis	Rajagopal, 2009; Zadoks and Fitzpatrick, 2009
	SDSD	C, G	Human and Bovine	Bovine mastitis, bacteremia	Koh et al., 2009; Park et al., 2012; Rato et al., 2013; Jordal et al., 2015
	SDSE	C	Human	Pharyngitis, bacteremia, septicemia, septic arthritis, endocarditis, meningitis	Brandt and Spellerberg, 2009
Salivarius	*S. salivarius*	K	Human	bacteremia, meningitis	Shewmaker et al., 2010
Sangui	*S. sanguinis*	Viridans	Human	Bacteremia, endocarditis, septicemia, meningitis.	Zhu et al., 2018
	*S. gordonii*	Viridans	Human	Bacteremia, endocarditis	Krantz et al., 2017

Pyogenic bacteria are responsible for causing purulent respiratory tract and skin infections; among these are pharyngitis, septicemia, necrotizing fasciitis, bacteremia, meningitis, pneumonia, septic arthritis, rheumatic streptococcal toxic shock syndrome, acute rheumatic fever/rheumatic heart disease, scarlet fever, as described in Table 1 and references therein. The actual burden of pyogenic infections is difficult to trace due to the variety of settings and the level of severity of the manifestations, which can go from mild symptoms as sore throat, escalating to severe, life-threatening, conditions. According to Carapetis and coworkers 2005 estimation, “there are at least 517 000 deaths each year due to severe *Streptococcus pyogens* diseases” (Carapetis et al., 2005).

Biofilm growth of streptococci has been investigated, but insights in the genetic origin of biofilm formation are limited. Although most pyogenic streptococci are able to develop biofilms, there is substantial heterogeneity in biofilm formation among individual strains (Konto-Ghiorghi et al., 2009; Trappetti et al., 2011; Marks et al., 2014a; Genteluci et al., 2015; Rosini and Margarit, 2015; Young et al., 2016; Alves-Barroco et al., 2019). Avoiding failure of antimicrobial therapy in the pyogenic streptococcal group requires an in depth knowledge of these singularities and the integration of chemical and physical methods to prevent/control/eradicate the biofilm formation. This review summarizes pyogenic streptococci biofilms with details on the constitution, formation, and virulence factors associated with formation.

## Biofilms Formation and Dispersal

**FIGURE 1:**
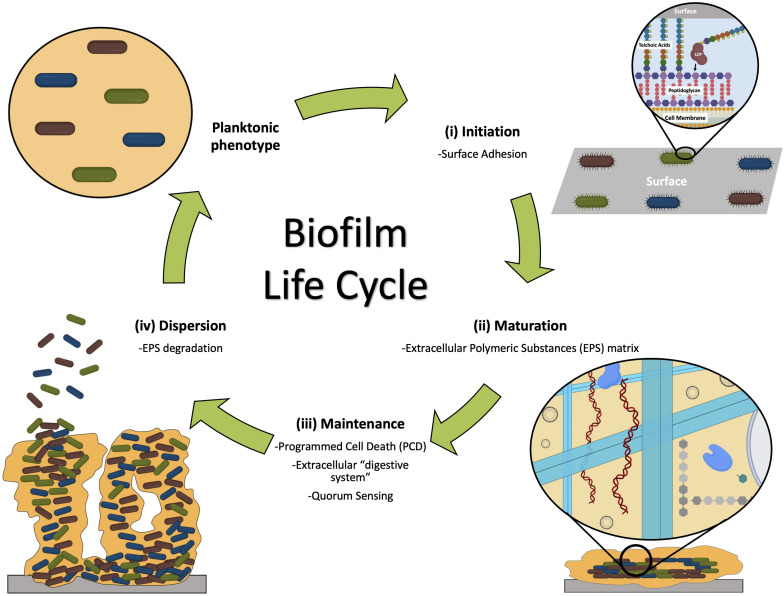
The three stages of biofilm formation and main events involved: (i) initiation, when bacteria attach to the surface; (ii) maturation, when microcolonies are formed, adhered to the surface and embedded by the EPS matrix; and (iii) dispersion, when some members of the community are released from the biofilm entering the planktonic phenotype. Maintenance of the biofilm ensures the community survival (Vasudevan, 2014).

Biofilm formation is a complex multi-step process, where adhesive and disruptive forces interplay (Jefferson, 2004; Chen and Wen, 2011; Young et al., 2016; Del Pozo, 2018). The formation of a biofilm is typically described in three stages: (a) initiation, where attachment occurs (which can be reversible and irreversible), (b) maturation, with microcolonies development, and (c) dispersal, where cells detach (Donlan, 2001; Garrett et al., 2008; Kumar et al., 2017; Jamal et al., 2018; Khatoon et al., 2018; Figure 1).

Several streptococci virulence factors have been described as associated with biofilms formation (Table 2). In the next sections we will highlight some of them.

**TABLE 2:** Streptococci virulence factors involved in biofilm formation.

Protein	Function and/or substrate	Species	References
Adhesion protein - AdcA	Cell adhesion; Zinc ion transport – Contributes to the infection process	*S. pyogenes*, SDSE, SDSD, SESZ, *S. pneumoniae, S. canis, S. agalactiae*	Moulin et al., 2016; Cao et al., 2018
Collagen-like protein 1 (*scl-1*)	Promotes adhesion and biofilm formation, decreases bacterial killing by neutrophil extracellular traps	*S. pyogenes, S. agalactiae, S. pneumoniae, and S. equi.*	Oliver-Kozup et al., 2011; Döhrmann et al., 2014; Lukomski et al., 2017
Collagen-like protein SclB (*sclB*)		S. *pyogenes, S. uberis*, SDSE, SDSD, *S. agalactiae, S. pneumoniae, and S. equi.*	Rato et al., 2011; Lukomski et al., 2017
FbaA	Fibronectin and laminin-binding protein	*S. pyogenes; S. parasanguinis*	Chen et al., 2020
FbaB/PFBP/PrtF2	Fibronectin and laminin-binding protein	*S. pyogenes*	Rohde et al., 2011; Brouwer et al., 2016; Šmitran et al., 2016
Fbp54	Fibronectin and fibrinogen-binding protein	*S. pyogenes*	Raynes et al., 2018
FbsA, FbsB	Fibronectin and fibrinogen-binding protein	*S. agalactiae*	Rosenau et al., 2007
FnbA, FnbB	Fibronectin-binding proteins	SDSD; SDSE	O’Neill et al., 2009; Alves-Barroco et al., 2018
GfbA	Fibronectin-binding proteins	SDSE	Lindgren et al., 1994; Oppegaard et al., 2018
Glyceraldehyde-3-phosphate dehydrogenase (*plr*)	Plasmin and Fn-binding protein	*S. pyogenes*, SDSE, SDSD, *S. suis, S. agalactiae, S. uberis*	Seifert et al., 2003; Rato et al., 2011; Reinoso, 2017
Hyaluronic acid (HA) capsule	Immune evasion upon colonization of host tissues; adhesion to the surface of the host cell	*S. pyogenes*	Falaleeva et al., 2014; Vyas et al., 2019
Laminin-binding protein (*lmb*)	Laminin-binding surface proteins	*S. pyogenes*, SDSE, SDSD, SESZ, *S. pneumoniae, S. canis, S. agalactiae*	Al Safadi et al., 2010; Rato et al., 2011
Lipoteichoic acid (LTA)	Adherence to epithelial cells of host Biofilm formation	S. *pyogenes, S. uberis*, SDSE, SDSD, *S. agalactiae, S. pneumoniae, and S. equi, S. gordonii*	Courtney et al., 2009; Rato et al., 2011; Shiraishi et al., 2016; Lima et al., 2019
M protein (emm)	Adherence to epithelial cells of host; Plasminogen, fibronectin and Fibrinogen-binding proteins; Ig-binding proteins	*S. pyogenes*, SDSE, SDSD, SDSZ, *S. canis*	Courtney et al., 2009; Rato et al., 2011; McNeilly and McMillan, 2014; Frost et al., 2018
M-like protein (*enn*) complexe			
Pili, fimbriae, fibrils	Biofilm formation Coaggregation, biofilm formation, phagocyte resistance; multiple substrates	*S. pyogenes*, *S. agalactiae*, SDSE, *S. pneumoniae*	Manetti et al., 2007; Proft and Baker, 2009; Rajagopal, 2009; Konto-Ghiorghi et al., 2009; Genteluci et al., 2015; Ma et al., 2017
R28	Promotes adhesion to host cells via direct binding to integrins	*S. pyogenes*, SDSE, SDSD	Rato et al., 2011; Weckel et al., 2018; Eraso et al., 2020
SfbI/PrtF1	Fibronectin-binding proteins; Ig-binding protein	*S. pyogenes*	Šmitran et al., 2016
SfbX	Fibronectin-binding proteins	*S. pyogenes*	Brouwer et al., 2016; Šmitran et al., 2016
HtrA	Serine protease; Degrades proteins in response to stress; bility to adhere to the extracellular matrix that enable adhesion to host tissues	*S. pneumoniae*, *S. pyogenes*, SDSD, SDSE	Lyon and Caparon, 2004; Kim and Kim, 2005
speB	Targets several different host proteins, namely interleukins, antimicrobial peptides, and components of the extracellular matrix	*S. pyogenes*	Doern et al., 2009; Roberts et al., 2010a; Connolly et al., 2011; Carothers et al., 2020
DNases	Degradation of NETs that are produced by the host’s immune system cells (like neutrophils)	*S. pyogenes, S. agalactiae*, SDSD, SDSE	Rato et al., 2010, 2011; Genteluci et al., 2015; Remmington and Turner, 2018
Biofilm regulatory proteins A BrpA	Biofilm formation, autolysis, and cell division	*S. pyogenes, S. agalactiae, S. mutans*, SDSD, SDSE	Bitoun et al., 2012, 2014; Alves-Barroco et al., 2018; Patras et al., 2018

### Regulatory Factors That Control Biofilm Development

Quorum-sensing (QS) is an intercellular communication system used by bacteria to control social behavior, intrinsically dependent on cell density. QS regulates both cooperation and competition within an interspecies bacterial community (Abisado et al., 2018). Several products, as proteases or toxins, are produced by individual cells but can be used by any member of the community, in a common good philosophy. QS has been implicated in all stages of biofilm development, namely by modulating initiation, maturation and dispersal (Parsek and Greenberg, 2005; Dickschat, 2010). Generally, QS relies on the production and sensing of extracellular signals. These systems are composed of two proteins and a signaling molecule; one of the proteins generates the signaling molecule, and the other protein acts as a sensor, activating the expression of other proteins. In the case of Gram-positive bacteria, the signal can be a linear or cyclic peptide, while the sensor is a two-component signal transduction system composed of a membrane-bound histidine kinase, and an intracellular response regulator (Kleerebezem et al., 1997).

In *Streptococcus*, the QS systems can be categorized into three main types: (i) Regulator of glucosyltransferase (Rgg), (ii) Streptococcal invasion locus (Sil), and (iii) LuxS/AI-2.

The Rgg family is composed of transcription regulators widely disseminated among Gram-positive bacteria, that respond to signaling peptides. These have been shown to modulate genetic determinants associated with virulence, competence, and biofilm establishment, maturation and dispersion in *S. pyogenes, S. pneumonia*, *Streptococcus gordonii, Streptococcus mutans*, and *Streptococcus intermedius* (Loo et al., 2000; Li et al., 2002; Petersen et al., 2004; Suntharalingam and Cvitkovitch, 2005; Mashburn-Warren et al., 2010; Junges et al., 2017). *S. pyogenes* contains four Rgg paralogs: RopB (Rgg1), Rgg2, Rgg3, and ComR (Rgg4) (Cook et al., 2013). RopB, the most studied Rgg, is required for transcription of the streptococcal pyrogenic exotoxin B (*speB*) gene that targets several different host proteins important for resistance (namely interleukins, antimicrobial peptides, and components of the extracellular matrix). The highest expression levels of speB are observed in the stationary phase, which suggests that gene activation by RopB requires high cell density (Cook et al., 2013). The role of the Rgg2 and Rgg3 systems to the *S. pyogenes* life cycle and pathogenesis has not been completely elucidated. Studies have demonstrated that these regulators respond to small hydrophobic peptides – SHP2 and SHP3, controlling differential gene expression and biofilm development. *S. pyogenes* Rgg2 is an activator of genes, while Rgg3 inactivate its expression; they compete for binding to the same regions on gene promoters, upstream of shp2 and shp3. In this way, Rgg2 and Rgg3 regulate the same function in antagonistic ways (Chang et al., 2011; Lasarre et al., 2012).

ComR-ComS (Rgg4) system has been reported to regulate streptococci competence genes in natural genetic transformation phenomenon. ComR – cytoplasmatic effector and ComS – the precursor of the competence pheromone, were first identified in *Streptococcus thermophilus* and *Streptococcus salivarius* (Gardan et al., 2013). The potential number of transformable *Streptococcus* was increased by the identification of orthologous in all available sequenced streptococci species (Fontaine et al., 2010).

It was experimentally proven that ComR-ComS system strongly induces ComX (also known as sigma factor σ^X^ or sigX) expression which leads to, consequently, natural DNA transformation in several species of the *Streptococcus* genus (Gardan et al., 2009; Fontaine et al., 2010; Khan et al., 2012; Marks et al., 2014b). In the early phase, *comX* rises in his net abundance inducing the X-state in cells, a transcriptional reprogramming (Claverys et al., 2006). When the cell reaches the late phase, σX associates itself with RNA polymerase core, making the target a specific target named σX-box, DNA-binding motif or Cin-box which regulates the expression of the regulon - typically consisting of fourteen critical late competence gene encoding the transformasome and many species-specific dispensable genes as the ones involved in the methylation of the exogenous ssDNA (Claverys and Martin, 2003; Petersen et al., 2004; Johnston et al., 2013).

Chemically defined medium with free amino acids and no complex oligopeptides seems optimal for the induction of *comX* by ComR-ComS. Some of the strains tested can be spontaneously transformable in similar conditions, but others being only transformable when growing as biofilm (Gardan et al., 2009; Fontaine et al., 2010; Marks et al., 2014a). In fact, in *Streptococcus*, the competence pheromones are produced in response (induced) to specific environmental stresses, including acidification of the medium, oxidative or temperature stress, mutagens, or nutrition stress (Fontaine et al., 2015).

In 2014, Marks et al. (2014a) reported the observation of *S. pyogenes* naturally competence *in vitro* in the form of biofilm structures, on epithelial cells and in biofilm colonization *in vivo* (BALB/cByJ mice). This was the first report of natural transformation in the pyogenic streptococcal group. ComR-ComS system orthologous genes are present in *S. agalactiae* and *S. dysgalactiae* subspecies, and it may, therefore, be a question of replicating the physiological model, like biofilm, to observe natural competence (Fontaine et al., 2015). ComR has dual functionality – in the growth of biofilms and natural transformation – which seems to point to a relation between these two processes (Marks et al., 2014a).

The Sil system is an important QS mechanism found in *S. pyogenes* that has been investigated for its potential role in biofilm formation through regulation of cell adhesion to surfaces (Young et al., 2016). The core *sil* system is encoded in a putative 15–17 kbps genomic island harboring six genes, *sil*ABCDE and *sil*CR, and a much higher GC content compared to the rest of the genome. The locus includes a two-component system (*silA* and *silB*), putative ATP-binding cassette transporters (silD and silE), and *silC* that together with *sil*C/R regulate the *sil* locus transcription (Jimenez and Federle, 2014). The *sil*C/R encode a small 41-aa pro-peptide, and this pheromone has been identified to be signal peptide associated with modulation of expression of various uncharacterized genes in *S. pyogenes* (Jimenez and Federle, 2014).

LuxS protein is present in several Gram-negative and Gram-positive bacteria and is responsible for the production of the autoinducer-2 (AI-2), which has been identified as a universal signaling molecule for interspecies communication. Previous studies have demonstrated that the LuxS/AI-2 system influence the expression of various virulence determinants, and biofilm development in *S.* species, including *S. gordonii, S. intermedius*, and *S. pyogenes* (Galante et al., 2015). It was found to be necessary for the proper formation of biofilms in *S. mutans*, by regulating the expression of glucosyltransferases (Merritt et al., 2003). The same system in *S. pneumoniae*, regulates the initial stages of biofilm formation through regulation of the LytA (autolysin) expression and pneumolysin (Vidal et al., 2011).

### Environmental Factors That Control Biofilm Initiation

Bacteria generally adopt the planktonic phenotype when nutrients are available namely, in a nutrient rich medium like the ones used in the laboratory. However, in nature, nutrients are not so abundant, and bacteria will then opt to grow in a phenotype where the growth rate is slower and less demanding metabolically, like the biofilm phenotype. This biofilm protects from dynamic environments and antimicrobial agents (Garrett et al., 2008; Olsen, 2015). The interplay between the planktonic phenotype and the biofilm state is regulated by several genetic determinants and transcription factors in response to environmental stimuli; these impact enzymatic and structural components of the cell that are required for biofilm development (Garrett et al., 2008; Kostakioti et al., 2013; Fiedler et al., 2015). Among the environmental factors, nutrient availability, temperature and pH variations, concentration of metals and osmolytes, redox potential, interaction with the host’s immune system are the most common. These factors affect bacterial cell properties, namely gene regulation and cell surface physicochemical characteristics, which may have a profound effect on cell–cell interaction and hence, on biofilm development (Garcia-Gonzalo and Pagán, 2015).

Several *in vitro* studies show that environmental factors influence the ability of streptococci pyogenic strains to form biofilms. Baldassarri et al. (2006) evaluated the influence of temperature and atmospheric conditions in the ability of *S. pyogenes* clinical strains to form biofilms. While temperature appeared to have no effect on the biofilm formation, there was a significant increase in biofilm formation under anaerobiosis (Baldassarri et al., 2006). An increased biofilm formation was also observed when *S. pyogenes* and *S. agalactiae* were grown in media supplemented with 1.5% (v/v) glucose as a result of medium acidification due to metabolism (Manetti et al., 2010; Rinaudo et al., 2010; Thenmozhi et al., 2011). D’Urzo et al. (2014) showed evidence that acidic pH and not glucose concentration is the environmental signal to *S. agalactiae* biofilm formation. However, some opposing results have been observed in several *in vitro* studies regarding the influence of pH in the biofilm formation by *S. agalactiae*. Some studies show an increased ability to form biofilms by *S. agalactiae* strains at pH 6.5 when compared to pH 4.2 (Kaur et al., 2009; Borges et al., 2012; Yang et al., 2012), by contrast, Ho et al. (2013) showed that low pH induced biofilm formation. These discrepancies can possibly be explained by the different strains intrinsic variability and ability to survive in an acidic environment. *In vivo*, exposure of *S. agalactiae* to the acidic environment of the vagina can be the signal sensed by the bacteria to grow in biofilm. From this perspective, changes in the growth of *S. agalactiae* strains were observed in a mouse model of vaginal colonization (Carey et al., 2014) and has also been reported in humans (Hansen et al., 2004). An increased ability to form biofilms *in vivo* was also observed by *S. pyogenes* and SDSE strains when compared to *in vitro* (Marks et al., 2014a; Genteluci et al., 2015). In this context, it is likely that apart from carbon sources, modified atmosphere, pH, and responses of the host’s immune system might trigger biofilm development *in vivo.*

Vajjala et al. (2019) characterized an *in vitro* model of *S. pyogenes* biofilm formation on mammalian cells that mimic a mouse model of human necrotizing fasciitis. The authors showed that the expression of secreted *S. pyogenes* streptolysins induced endoplasmic reticulum stress in the host. *In vivo*, the streptolysin null mutant is attenuated in biofilm formation and bacterial spread, revealing an important role of streptolysin in endoplasmic reticulum stress and the association with the formation of biofilms and necrotizing fasciitis disease progression (Vajjala et al., 2019).

### Attachment Factors Involved in Initiation

Biofilm development starts when individual cells adhere/attach to abiotic or biotic surfaces. Attachment is mediated through several structures and molecules present at the surface of the bacterial cells, like motility elements as the flagellum or proteinaceous structures like the pili (type-IV pili), and other elements like lipopolysaccharides, TAs, surface proteins, and extracellular proteins (Schumacher-Perdreau et al., 1994; Hussain et al., 1997).

In *S. pyogenes*, attachment is suggested to occur in a two-step process with different molecules coming into play. The first step is dependent on lipoteichoic acids (LTA); in the second step bacteria cells adhere to specific receptors on the host cell (namely human) via bacterial surface components known as microbial surface components recognizing adhesive matrix molecules (MSCRAMMS), which include M protein, fibronectin-binding protein, serum opacity factor, etc. (Morath et al., 2005; Weidenmaier and Peschel, 2008; Rohde and Cleary, 2016).

While the first step is a very dynamic process, with an on/off kinetic effect, and relying on hydrophobic, ionic, and electrostatic forces responsible for an initial attachment, the second step of adhesion is more specific and complex since the strong affinity between several MSCRAMMS and host cells promote irreversible and species-specific interactions. *S. pyogenes* can adhere to human cells by interacting with different MSCRAMMS simultaneously, which strengthens biofilm initiation but hampers the detection of streptococcal adhesins (Šmitran et al., 2016).

#### Lipoteichoic Acids

Teichoic acids constitute the main class of anionic glycopolymers that are composed of phosphodiester-linked polyol units and are divided into two types: LTAs, and wall teichoic acids (WTAs; Armstrong et al., 1958; Ward, 1981; Brown et al., 2013). The difference between the two types is their connection to cell, with WTAs being covalently attached to the peptidoglycan, and LTAs are anchored in the cell membrane. LTA has been characterized in most Gram-positive bacteria including *Staphylococcus aureus*, *S. agalactiae, S. pyogenes*, and *Lactobacillus plantarum* (Kang et al., 2016). WTA has been less characterized in the pyogenic group and detailed studies revealing its importance are missing in the literature. With the growing number of pyogenic isolates available, future developments are expected in this topic. Teichoic acids biosynthesis is a complex multistep mechanism, and different WTAs/LTAs compositions and modifications are observed in different species (Poxton, 2014). The main chain of these biopolymers can be further modified by D-alanylation, by adding D-alanyl esters, and glycosylation, by adding mono- or oligosaccharides. These modifications impact several functions of the TAs.

**FIGURE 2:**
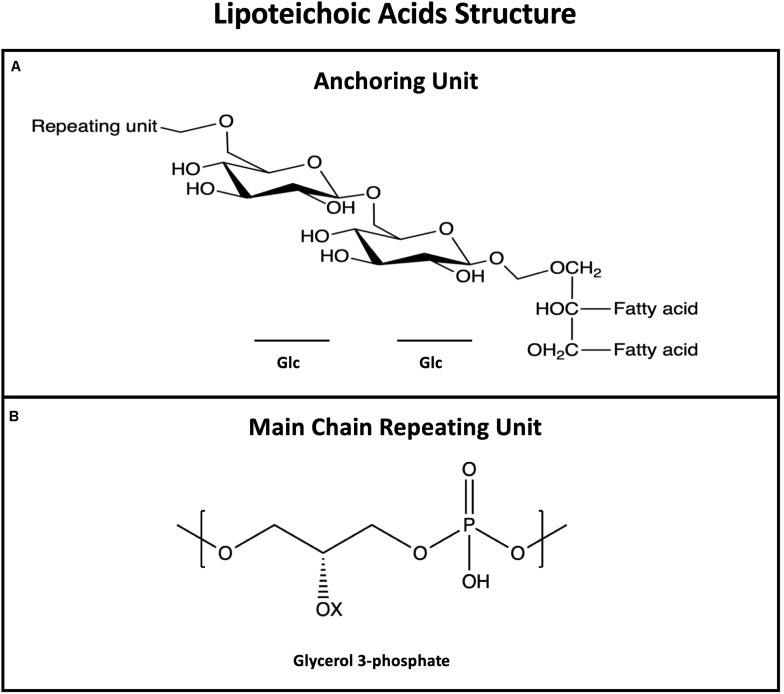
General structure of lipoteichoic acids (LTAs) showing the membrane anchoring moiety **(A)** and the most common repeating unit **(B)**. LTAs repeating unit is more conserved than WTAs; Opposite to WTAs, LTAs are not bound to peptidoglycan but anchored to the membrane via the fatty acid.

Lipoteichoic acids are long, linear, anionic glycopolymers of phosphodiester-linked poly-glycerol phosphate (poly-GroP) repeating units, anchored to the plasma membrane of the cell through a glycolipid (Figure 2; Morath et al., 2005; Weidenmaier and Peschel, 2008; Shiraishi et al., 2016). LTAs are known to be major players in biofilm formation – studies estimate that 60% of initial adherence to epithelial cells is mediated by LTA (Šmitran et al., 2016) – however, other physiological roles are attributed to this amphipathic molecule: control of autolytic enzymes, maintain membrane integrity, cation homeostasis, and ions/nutrients trafficking (Fischer, 1988; Neuhaus and Baddiley, 2003; Poxton, 2014).

The amphipathic nature of LTAs is crucial for pyogenic bacteria to surmount the repulsive electrostatic forces between bacteria and the attaching surface; LTA hydrophobic moieties are made accessible to the bacteria surface when LTA form stable complexes with surface proteins (see description of M proteins in the next section) while the anionic character achieved by the presence of phosphate and amino groups is important for interaction with cell receptors (as type I macrophage scavenger receptor) mediating bacteria-host adhesion (Dunne et al., 1994).

Several studies suggest that among the *S. pyogenes* strains the formation of complexes with M proteins provide anchoring LTA on the surface contributing to hydrophobicity and to biofilm formation (Courtney et al., 2009). Protein M is practically ubiquitous among *S. pyogenes* isolates, being expressed on the surface of the bacterial cell. Studies show that M proteins provide adhesion to different human cell lines, and the tropism for different cells demonstrates the heterogeneity of this protein (Tylewska et al., 1988; Caparon et al., 1991; Rohde and Cleary, 2016). It has also been demonstrated that M protein mediates initial surfaces interactions during biofilm formation (Cho and Caparon, 2005; Courtney et al., 2009). It was suggested that interactions between M protein of *S. pyogenes* and LTA expose the ester fatty acids of LTA, increasing bacterial hydrophobicity providing LTA–host cell interactions (Courtney et al., 2009). This structural conformation of the LTAs fosters biofilm development since it allows auto-aggregation and surface adhesion (Cho and Caparon, 2005; Lembke et al., 2006; Courtney et al., 2009; Young et al., 2016), which consequently influences the ability of some members of this species to form biofilms. This hypothesis is also supported by studies with M-protein defective mutants that showed a decrease in biofilm development regarding wild type strains (Cho and Caparon, 2005; Courtney et al., 2009). On the other hand, the studies by Courtney et al. (2009) suggest that the M-protein–LTA interaction may be specific to strains expressing only one M protein family member.

Protein M homologs (Mrp and Enn proteins) genetically and functionally related are present in most *S. pyogenes* strains. Despite considerable similarities among M, Mrp, and Enn proteins, the M-like proteins remain less studied. The current state of knowledge for M, Mrp and Enn proteins, is based on studies on function and immunogenicity, interactions between M-like proteins and host ligand proteins, and analysis of the genetic data supporting these interactions (reviewed in Frost et al., 2018). Overall, the similarity of protein M, the M-like proteins have been shown to be involved in the maintenance of LTA which may aid in the formation of biofilms and in colonization of the oropharynx (Beachey et al., 1983; Courtney et al., 2009). M-like proteins have been identified among SDSE strains (McNeilly and McMillan, 2014); however, their role in the formation of biofilms is not yet determined.

#### Streptococcal Pili

Pili (or fimbriae) are long filamentous structures that are extending from the surface of several bacterial cells, including SDSE (Genteluci et al., 2015; Ma et al., 2017), *S. pyogenes* (Manetti et al., 2007) and *S. agalactiae* (Konto-Ghiorghi et al., 2009; Rajagopal, 2009). Many functions can be attributed to pili; besides adhesive structures, they have been implicated in gene transfer, biofilm development, host cell invasion, twitching motility, and biofilm development, in the latter case by stimulating bacterial aggregation and attachment to the surfaces of host cells (Proft and Baker, 2009; Rohde and Cleary, 2016).

*Streptococcus pyogenes* pilus proteins have a C-terminal LPTXG-like motif attached to the host cell wall (Mora et al., 2005) and in pilus-defective mutants, a reduced biofilms formation ability was observed in different surfaces (Manetti et al., 2007). The role of pili in *S. pyogenes* virulence has also been evaluated *in vivo* in a humanized mouse by the deletion of AP1 gene, encoding the ancillary proteins (Lizano et al., 2007). This clearly shows the importance of pili in the initial stages of host colonization by *S. pyogenes* (Danne and Dramsi, 2012). In another study it was observed that pilus promote the recruitment of immune system cells, mainly neutrophils that provide neutrophil extracellular traps (NETs), which results in the decrease of *S. pyogenes* virulence in murine models (Crotty Alexander et al., 2010).

*Streptococcus agalactiae* strains encode two genomic islands, pilus islands-1 (PI-1) and -2 (PI-2), which includes the two variants PI-2a and PI-2b in separate loci and both flanked by direct repeats of conserved genes, where all genes responsible for pilus machinery are located. These genomic islands harbor three genes encoding protein essential for pilus assembly and accessory proteins (AP1 and AP2). Besides these, there are also two genes encoding sortase responsible for polymerizing the protein chains and cell wall attachment (Dramsi et al., 2006; Rosini et al., 2006). The importance of the genomic islands for bacteria–host cell interaction and biofilm formation in abiotic structures was revealed by previous studies with isogenic mutants lacking pilus 2a structures or the sortase enzymes demonstrated the role of pili in the interaction of bacteria with the host cell and biofilm establishment on abiotic surfaces (Rinaudo et al., 2010). Rinaudo et al. (2010) also used antibodies directed against the pilus 2a and its ancillary protein, or antibodies against small ancillary proteins present at the base of the pilus. The results showed that the first repressed biofilm development in a dose-dependent manner while the latter had no effect, suggesting that PI-2a pili are more relevant for biofilm adherence and formation in these bacteria then the other components (Rinaudo et al., 2010).

The presence of fibrillary-like structures was also observed on scanning electron microscopy in biofilm-grown SDSE strains. The increased expression of coding genes was observed in strong biofilm-producing strains (Genteluci et al., 2015). Oppegaard et al. (2017) showed that genetic alterations in the pilus-region (also known as the FCT-region – Fibronectin and Collagen binding, and T-antigen) were associated with bacteria’s propensity to adhere to different surfaces, which could also be related to the expression of a wide range of adhesins putatively encoded in this region, including proteins predicted to adhere to collagen and fibronectin. Interestingly, although they are genomic closely related, the presence of fibrillary-like structures was not observed in SDSD strains. Probably, the absence of fibrillary-like structures in this subspecies can be compensated by the expression of another protein with similar functions (Alves-Barroco et al., 2019).

#### Adhesins

The strategies for adhesion are complex and variable, and the expression of specific-adhesin is considerably modulated by microenvironment conditions (Rohde and Chhatwal, 2013). Several fibronectin-binding proteins are extensively expressed in streptococci, with different binding properties and affinities, giving rise to a great variety of protein–protein interactions; in some specific strains the fibronectin-binding proteins have high affinity to soluble fibronectin, whereas other strains require immobilized fibronectin for binding. Due to their importance, eleven different fibronectin-binding proteins are expressed in *S. pyogenes*, including SfbI/F1, FbaA, FbaB, FBP54, protein F2, serum opacity factor, and several M proteins (Rohde and Cleary, 2016). Streptococcal fibronectin-binding protein I (SfbI or protein F1) takes part in the adhesion to different surfaces and recently was found to bind to host collagen facilitating the bacterial aggregation, colonization, and evasion to the host immune system (Dinkla et al., 2003). Fibronectin-binding proteins have also been described in SDSE, including FnbA, FnbB, FnB, and fibronectin-binding protein A (GfbA). These proteins provide streptococcal adherence to human skin fibroblasts, consequently contributing to biofilm development *in vivo* and persistence of infection (Collin and Olsén, 2003; Brandt and Spellerberg, 2009).

Adhesion to different surfaces are largely conditioned by the physicochemical substratum characteristics, including surface charge and hydrophobicity (Donlan and Costerton, 2002). These characteristics could be further modified by other environmental factors, such as temperature variations, and sub-inhibitory concentrations of antimicrobials (Garcia-Gonzalo and Pagán, 2015; Ranieri et al., 2018; Šmitran et al., 2018). As expected, bacterial cells with hydrophobic characteristics adhere to hydrophobic surfaces, cells with hydrophilic properties adhere to hydrophilic surfaces. Additionally, hydrophobic cells are more able to attach to surfaces than hydrophilic (Garcia-Gonzalo and Pagán, 2015). During initiation, the attachment step is followed by a so-called “accumulative phase,” where the cells start to adhere to each other forming clusters (microcolonies). Cell–cell adhesion mechanisms mediate this multistep process through different factors. One of these factors is the glucosamine-based polysaccharide intercellular adhesin (PIA), or poly-N-succinylglucosamine (PNSG). The biosynthesis of PIA starts with the expression of the IcaA protein, the first product of the ica operon (Cue et al., 2012). Orthologous genes of the IcaA protein are found in two *S. agalactiae* strains (data from BioCyc database). The IcaA protein is a N-glycosyltransferase – glycosyltransferases are responsible for the production of exopolysaccharides necessary for the production of the EPS matrix and establishment of microcolonies, and are found in *S. mutans* (Koo et al., 2010). Recently, Matysik and Kline (2019) proposed a microcolony-independent mechanism for the *S. pyogenes* clinical strain, JS95. In this alternative mechanism, instead of forming microcolonies the cells will sediment and attach to the surface, with the capsule being of crucial importance. The role of the capsule in biofilm formation has been elusive in the past, with its presence being associated with biofilm inhibition. The same study suggests a dual role for the capsule that is growth stage dependent. At the early exponential growth, the capsule will mask the surfaces adhesins, inhibiting biofilm formation, while at later growth phases the capsule will promote biofilm formation (Matysik and Kline, 2019).

### Disruptive Factors and Biofilm Dispersal

The presence of disruptive factors is of extreme importance for the proper development of the biofilm. These factors help the biofilm to reach its mature structure, with the creation of water channels and cavities; without these features the biofilm structure will be impaired at several levels, as the EPS matrix will not have the right properties. Also, factors with hydrolytic properties play important roles in the turn-over of the adhesive factors, allowing them to be replaced. This way the structure of the biofilm is maintained. Another very important function of the disruptive factors is their involvement in the return of the cells to the planktonic phenotype (dispersal stage). Proteases, together with DNases, are responsible for the degradation of the polymeric matrix of biofilms its tight regulation ensures the maintenance of the biofilm structure since high levels of these enzymes lead to disruption of the 3D architecture of the biofilm and ultimately, to its dispersion (Fiedler et al., 2015).

#### Proteases

The streptococcal pyogenic exotin B (SpeB), a cysteine protease, is known to play a substantial role in *S. pyogenes* virulence. Studies show that SpeB can destabilize the extracellular matrix of the host cell, complement system molecules, immunoglobulins, interleukin, antimicrobial peptides, and serum protease inhibitors (Hytönen et al., 2001). SpeB is also responsible for the ability of *S. pyogenes* to colonize host cells since it is involved in adhesion to glycoproteins. However, with high levels of active SpeB the biofilm enters the dispersion phase and its development is prevented (Doern et al., 2009). Several transcriptional regulators associated to the transcription of *speB* have also been found to regulate the formation and maturation of biofilms in *S. pyogenes*. RopB (Rgg family) and the sugar metabolism regulator CcpA are positive regulators that directly interact with promoter of the *speB* gene (Chang et al., 2011), while the transcriptional regulator Srv down-regulates this gene. When Srv is inactivated, an increase of the expression of SpeB is observed and the consequence is biofilm dispersal (Doern et al., 2009; Roberts et al., 2010a; Connolly et al., 2011). Roberts et al. (2010a) demonstrated that Srv regulates SpeB transcription in the initial step of the adhesion, that is, Srv represses SpeB, which allows for biofilm development. In response to external environmental signals, Srv decreases this repression, thereby providing SpeB production and consequent biofilm dispersion with the colonization of new sites. The response regulator CovR of the CovRS two-component system interacts with *speB* promoter, acting as a transcriptional repressor.; again, repression of *speB* by CovR allows the biofilm development (Doern et al., 2009; Roberts et al., 2010b; Connolly et al., 2011). CodY is another regulator associated with biofilm formation, involved in response to nutrient deprivation. *in vitro* studies with CodY deletion mutants demonstrated a reduction in the ability of biofilm formation by *S. pyogenes* (Fiedler et al., 2015).

The high-temperature requirement protein A (HtrA) is a protease widely distributed among streptococci that contains a transmembrane domain responsible for anchoring the enzyme to the cell surface. As SpeB, HtrA degrades other proteins in response to stress. Homologs of the HtrA identified in other Gram-positive bacteria degrade abnormal proteins in response to adverse environmental conditions (Kim and Kim, 2005). An equivalent role of this protein suggested for streptococci since the deletion of *htrA* in *S. mutans* decreased its ability to tolerate environmental stress (Diaz-Torres and Russell, 2001), and altered biofilm appearance, becoming more granular than wild type (Biswas and Biswas, 2005). Additionally, in *S. pyogenes*, the expression of virulence determinants was affected (Lyon and Caparon, 2004). In addition to the proteolytic properties of these enzymes, they share an ability to adhere to the extracellular matrix that enable adhesion to host tissues (Lyon and Caparon, 2004; Kim and Kim, 2005).

#### Extracellular DNases

Despite its abundance among *Streptococcus* species, the biologic role of e-DNA in biofilm development is not completely understood. The DNases of the *S. pyogenes* are the best characterized so far; six of the genes encoding DNases in this species were found in prophage regions (sda1, sda2, spd1, spd3, spd4, and sdn) and two other genes were found in the chromosome (spnA and spdB). Homologs of *S. pyogenes* DNases associated with prophage and chromosomally encoded have been found in other streptococcal species, as SDSD and SDSE (Rato et al., 2010, 2011; Remmington and Turner, 2018). The existence of extracellular nucleases in *S. agalactiae* has been proposed since the 1980’s. The major DNase in this species is encoded by the gene gbs0661, and the protein is named Nuclease A (NucA; Derré-Bobillot et al., 2013).

The focus in studying streptococcal DNases is the degradation of extracellular niches that are produced by the host’s immune system cells (like neutrophils), to capture and eliminate bacteria. Neutrophils secrete long, entangled, e-DNA chains called Neutrophil extracellular traps (NETs) that are enriched with antimicrobial molecules, small peptides and proteases. These traps imprison and degrade invasive cells. The DNA-based structure of the NTEs is not affected by the host proteolytic activity but can be hydrolyzed by the bacterial eDNases, releasing bacteria from the trap (Brinkmann and Zychlinsky, 2012; Remmington and Turner, 2018).

Streptococci DNases may also be involved in managing the biofilm since eDNA is one of the important components of the EPS matrix (Sharma and Pagedar Singh, 2018). By cleaving eDNA chains, DNases are involved in QS, regulation of biofilm formation, disruption of competitive biofilms and bacterial clearance (Sharma and Pagedar Singh, 2018).

Studies show that DNase is effective in removing Streptococcal biofilms on several substrates (Genteluci et al., 2015). The regulatory mechanism that control the protease SpeB, CovR/CovS, Rgg, and CodY, together with the systems PerR and Ihk/Irr, regulate the expression of DNases (Sumby et al., 2006; Anbalagan and Chaussee, 2013; Wang et al., 2013). Depending on the DNase the system might up- or down-regulate its expression. Yet, further research is required to fully elucidate the mechanism of regulation of DNases (Remmington and Turner, 2018).

The same disruptive forces necessary for the proper maturation of the biofilm are crucial for the detachment of bacterial cells from an established biofilm. This dispersal step usually leads to a modification in gene expression and allows the colonization of bacterial pathogens in other infection spots, and for this reason, the development of new biofilms, leading to the systemic diffusion of infection (Donlan, 2001; Kaplan, 2010; Kostakioti et al., 2013; Jamal et al., 2018).

The biofilm dispersion can be initiated by the bacterial community, called active dispersal, or by external forces – fluid shear, bacteriocin, and human intervention – called passive dispersal (Kaplan, 2010; Kostakioti et al., 2013). The mechanisms in which pyogenic streptococcal actively regulates biofilm dispersal is not yet completely understood. Overall, the presence of DNA and protein in the matrix of pyogenic streptococcal biofilms suggest that uncharacterized DNase or protease may participate in the regulation of biofilm dispersal (Doern et al., 2009; Genteluci et al., 2015; Young et al., 2016; Guilhen et al., 2017; Alves-Barroco et al., 2019).

Studies suggest that the streptococcal regulator of virulence (Srv) and the streptococcal pyrogenic exotin B (SpeB) play a significant role in the dispersion of *S. pyogenes* biofilm. *in vivo* and *in vitro* studies demonstrated that the deletion of *srv* coupled with an increase of SpeB results in decreased biofilm production. When the *srv* is active, SpeB levels increase, promoting degradation of components in the biofilm matrix allow biofilm cells to return to a planktonic form (Doern et al., 2009; Roberts et al., 2010a, b; Connolly et al., 2011).

### EPS Matrix

**FIGURE 3:**
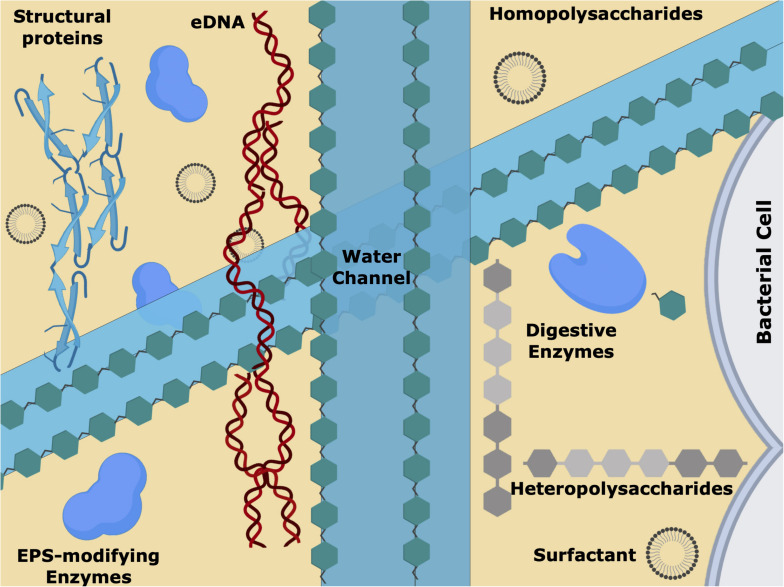
Schematic representation of the extracellular polymeric substances (EPS) matrix, showing the biofilm water channels, surfactants, the short and long sugar chains, the different proteins/enzymes and extracellular DNA (eDNA). This intricate 3D net of biomolecules is responsible for the chemical/physical and mechanical characteristics of the biofilm, allowing cell–cell communication, nutrients/gas diffusion and antimicrobial tolerance.

The biofilm is a dynamic and heterogeneous environment that allows bacterial cells to reach homeostasis, and to use all available nutrients (Sutherland, 2001; Flemming and Wingender, 2010). The EPS matrix composition is extremely important for the properties of the biofilm since it offers cohesion and a three-dimensional scaffolding structure that holds the microbial cells close and provides mechanical integrity to the biofilm (Figure 3) (Wilking et al., 2011). Overall, the nature of the biofilm matrix will depend on the microbial cells present, their physiological status, the nutrients available, and the environmental/physical conditions (Costa et al., 2018; Florez Salamanca and Klein, 2018).

Whether a microbial biofilm will form on biotic/abiotic surfaces or not, is a consequence of the EPS matrix formed and how it responds to stress. To ensure spreading, survival, and adaptation to changing environments, the biofilm creates spatial heterogeneity of extracellular components and generates different types of forces: (i) elastic tension, due to polymeric chains with weak hydrogen bonding; (ii) viscous damping due to friction of polymeric compounds and hydrogen bond breakage, and (iii) alignment of the polymers in the shear direction. Since biofilms are composed by cells and a polymeric gel, they have features of both solids and liquids and hence are considered an active viscoelastic material, exhibiting time-dependent response to mechanical stress (Wilking et al., 2011). Such properties change with increasing temperatures (Sutherland, 2001; Garrett et al., 2008; Flemming and Wingender, 2010).

The main component of biofilm is water. Representing 97% of the EPS matrix (although, as with all aspects of biofilms, this depends on the system under examination), the water establishes a hydrated environment that protects the bacterial community from desiccation. The EPS is an intricate 3-D network of long polymeric chains of sugars and e-DNA, decorated with enzymes and structural proteins, and filled with water. Acting as solvent, the water content dictates viscosity, mobility, and mechanical response; water is organized within the fine structure of the biofilm, and while filling the internal channels of the biofilm, mediates nutrients transport throughout the colonies, potentially mitigating starvation (Sutherland, 2001; Lu and Collins, 2007; Flemming and Wingender, 2010; Kumar et al., 2017; Jamal et al., 2018). Increasing evidence suggests that the extracellular matrix of pyogenic streptococcal biofilms is rich in proteins and, as in other organisms, these play an essential role in biofilm development, maintenance, and spreading (Genteluci et al., 2015; Rosini and Margarit, 2015; Young et al., 2016; Alves-Barroco et al., 2019). Within the proteinaceous group are the structural proteins and the enzymes, which can be further divided into (digestive) enzymes and EPS modifying enzymes (Flemming and Wingender, 2010).

Enzymes present in the EPS matrix can degrade it; a biofilm is a microconsortium of microorganisms and enzymes participate in the extracellular digestive system, by supplying low molecular mass products arising from the breakdown of biopolymers. This metabolic activity contributes decisively for the maturation of the biofilm by forming channels and pores that allow diffusion, but also for the detachment of bacteria from the biofilm during the dispersal step. Usually, these enzymes are maintained close to the cells through interactions with the exopolysaccharide chains, enhancing nutrients uptake, and protecting the enzymes from denaturation and proteolysis (Schumacher-Perdreau et al., 1994; Wingender et al., 1999).

The EPS modifying enzymes help in the maintenance of the EPS matrix but also in mechanisms of proliferation and adaptation, by targeting different elements of the matrix to be modified. Epimerases modify the structural conformation of the polysaccharides, influencing the intermolecular interactions within the biofilm and its 3D architecture (Whitfield et al., 2015). Hydrolases and transferases and add or remove functional groups as acetyls, glyceryls, pyruvyls, lactyls to the exopolysaccharide chains, controlling the polymer’s electrostatic potential and solubility. These modifications protect bacteria cells against ROS produced by host immune cells, antimicrobial peptides, bacteriocins produced by competing bacterial, ultimately contributing to fixation, and establishment of colonization and infection. Acetylation has been shown to play a critical role in *S. pneumoniae* – the presence of acetyl-decorated polysaccharides in *S. pneumoniae* clinical isolates seemed to be advantageous for the bacteria to resist recognition by innate immune mechanisms, making them more virulent than modification-free biofilms (Vuong et al., 2004; Rajam et al., 2007; Melin et al., 2010).

Structural proteins have a pivotal role in keeping the EPS matrix intact (Lasa and Penadés, 2006). Extracellular lectins, which are carbohydrate-binding proteins, are bridging the polysaccharide chains and cell surface-associated proteins, acting as cross-linking elements. Amyloid fibbers are also extensively formed in biofilms; long, ordered, and H-bonded beta-sheets of proteins or peptides self-assemble to form functional fibbers, that contribute to biofilm architecture and integrity (Fowler et al., 2007; Besingi et al., 2017). Furthermore, these low-energy protein structures, with a tensile strength comparable to steel, are resistant to degradation by detergents and proteases (Higgins and Novak, 1997; Van Schaik et al., 2005; Otzen and Nielsen, 2008). *S. mutans* is responsible for biofilm formation in the oral cavity, and amyloid fibbers are detectable in dental plaque. Inhibition of the biofilm is obtained using inhibitors of the amyloid-fibril forming proteins as adhesin P1 (Ag I/II, PAc) or wall-associated protein A (WapA; Oli et al., 2012; Besingi et al., 2017).

The EPS matrix is also composed of long chains of homo/heteropolysaccharides. The type of sugar residues, the net charge, the presence of organic functional groups or inorganic substituents (Ca^2+^ and other ions), and the internal glycosidic bonds present, affect the physical and, as a consequence, the biological properties of the matrix. Bacteria produce different polysaccharides along the “life-cycle” of the biofilm, and these polysaccharides have competent ability to interact with themselves, the biotic/abiotic substratum, and the other players of the biofilm arena, gaining an important role regarding function and structure. This might explain why many bacteria require exopolysaccharides to form a mature biofilm. Aggregative polysaccharides are essential for initial attachment, microcolony and macrocolony formation, and detachment or disassembly. Protective polysaccharides create a barrier that obstructs the entrance of antimicrobial agents, giving microcolony time to respond to the stimuli by upregulating protective genes. Capsular polysaccharides are covalently bound to the bacteria cell surface and grant chemical and structural variability to the cell and is one of the most effective strategies to deceive the host immune system (Wessels, 2016).

Polysaccharides of the EPS are often covalently bound to lipids. These lipopolysaccharides are slightly negatively charged, shield off the hydrophobic antimicrobial agents, and form a protective gelatinous layer at the bacteria surface.

Extracellular DNA is a structural element of the EPS and a source of genetic material. It arises from cell lysis, but also from specific mechanisms (autolysis, active secretion mechanisms, or formation of membrane vesicles) that allow DNA damage repair and HGT, and provide symbiotic competitive advantages to the community (Ibáñez de Aldecoa et al., 2017). The eDNA releasing mechanisms are modulated by quorum sensing – a cell-to-cell communication system that allows a coordinated response of the microorganisms. This is the case for *S. pneumoniae*, where a certain level of cell density triggers the lysis of a subpopulation, allowing the release of genomic DNA (for details, see Ibáñez de Aldecoa et al., 2017 and references therein). Genetically encoded systems have been found to control programmed cell death for the release of bacterial DNA (Bayles, 2007). In 2007, Bayles (2007) described a system that works based on a holin-like (CidA) and antiholin-like (LrgA) proteins, responsible for regulating programmed cell death (PCD). Holins are membrane proteins that promote lysis and antiholins counteract holins. PCD is extremely important for biofilm formation, and this regulatory system is virtually ubiquitous to all bacteria. It is proposed to be associated with antibiotic resistance: like what happens in drug-resistant cancers when Bcl-2 is overexpressed, it seems that in biofilms, cells will resist to death because the system will be preventing cell death. Studies where cidA or lrgA genes were deleted or mutated strongly suggest this point.

Recently, it was demonstrated, the capacity of *Streptococcus dysgalactiae subs. dysgalactiae* to produce biofilms and the multifactorial nature of their composition (Alves-Barroco et al., 2018). Confocal laser scanning, fluorescence microscopy and scanning electron microscopy were used to analyze the SDSD biofilm structure. The results achieved demonstrated that there are differences in biofilms produced among different SDSD strains, and in some cases, the presence of mucus-like extracellular material covering the cell surface was observed in the biofilms. This material corresponded to eDNA and proteins since in the presence of proteinase K, biofilm production by SDSD strains is inhibited (Alves-Barroco et al., 2019).

Doern et al. (2009) examined *S. pyogenes* strains from different clinical origins and showed that DNA and proteins are the major structural components of the biofilm, while carbohydrates had a modest role (Doern et al., 2009). This is in contrast to SDSE, which requires the presence of several different polysaccharides (Genteluci et al., 2015). Also, adding a carbohydrate oxidant as sodium metaperiodate to SDSE biofilm indicated the presence of an exopolysaccharide, similarly to what was observed for different biofilms of *S. mutans* (Liao et al., 2014) and *S. intermedius* (Nur et al., 2013).

The importance of eDNA was also demonstrated by disruption of the biofilm structure of SDSE strains after treating with DNase I. The authors suggest that eDNA is essential for the initial stage of adhesion and the biofilm development. The secondary role of proteins in the biofilm structure was proposed by the low protein content, 12.14%, and its disruption upon treatment with proteases (Genteluci et al., 2015).

## Clinical and Veterinary Relevance of Pyogenic Biofilm

The significance of this bacterial phenotype in clinical settings is often underestimated (Hall-Stoodley et al., 2004; Chen and Wen, 2011; Kumar et al., 2017). Several different surfaces in clinical environments are prone to develop biofilms, consequently increasing the risk of infection (Garnett and Matthews, 2012; Garnett et al., 2012). Some characteristics of the human body as shear forces caused by teeth, blood pressure, or the innate immune system, actually trigger the bacterial cells to adopt the biofilm phenotype, as they resemble some of the stimuli that induce biofilm formation in challenging environments (Stewart and Costerton, 2001; Jefferson, 2004; Gupta et al., 2016).

The main requirements for successful infection of different tissues is the ability of bacteria to adhere and to remain attached to the host cells. In these environments, the development of biofilm enhances resistance to host defenses and to nutrient privation, improving bacterial survival (Hall-Stoodley et al., 2004; Melchior et al., 2006; Kumar et al., 2017). From a clinical point of view, there is a considerable relationship between the ability to form biofilms and resistance to conventional antibiotics (Sharma et al., 2019). According to the National Institute of Health, in humans, biofilms account for up to 80% of the total bacterial infections, including endocarditis, periodontitis, sinusitis, meningitis, osteomyelitis, chronic wounds, and prosthesis and implantable devices related infections (Khatoon et al., 2018). In many of these cases, infection arises from implantable medical devices, such as catheters, implants, and implantable electronic devices (Khatoon et al., 2018; Narayana and Srihari, 2019; Pelling et al., 2019) that become contaminated with bacteria, usually biofilms of staphylococci, streptococci, Gram-negative bacteria, and fungi (Kokare et al., 2009; Marks et al., 2014b; Rosini and Margarit, 2015; Gomes et al., 2016; Young et al., 2016; Castilho et al., 2017; Stewart and Bjarnsholt, 2020).

*Streptococcus pyogenes* biofilm was first detected in the skin (Akiyama et al., 2003) and accepted to occur in other sites of infection (Lembke et al., 2006; Swidsinski et al., 2007). Pyogenic bacteria can integrate the host natural microbiome or form its own biofilm during infection. Apart from person to person contact, contaminated airborne droplets are the most common way of transmitting the pathogen within humans. Bacterial colonization occurs in mucosal membranes of the oropharynx and non-intact skin; for disease to develop, bacterial cells first adhere and later internalize the host cells (Fiedler et al., 2015). Very recently, Siggins showed that in severe invasive infections, *S. pyogenes* reach the bloodstream using efferent postnodal lymphatic vessels through sequential draining lymph nodes (Siggins et al., 2020). The authors also showed that while traveling form the primary site of infection, the bacteria remains extracellular. Self-healing or severe invasive infections are dependent on several different factors, namely the formation of biofilm. In 2013, Fiedler et al. (2013) demonstrated that *S. salivarius* and *Streptococcus oralis* – abundant species in the oral cavity – can form mixed-species biofilm with *S. pyogenes*; in this case, *S. pyogenes* is in the upper layer. The biofilm phenotype can be correlated with asymptomatic carrier persons, recurrent infections, systemic dissemination of the infection and antibiotic failure (Fiedler et al., 2013, 2015).

In veterinary settings, the production of biofilms can significantly affect the effectiveness of the treatment of bovine mastitis. Besides increased resistance to different antibiotics, the biofilm promotes adherence and colonization of mammary tissue (Wallis et al., 2018). In bovine mastitis, the most prevalent species of the pyogenic group are *S. agalactiae*, *Streptococcus uberis*, and SDSD (Rato et al., 2013; Rosini and Margarit, 2015; Pang et al., 2017; Klaas and Zadoks, 2018; Kaczorek et al., 2017; Tomazi et al., 2018).

*Streptococcus uberis* and SDSD were initially classified as environmental, meaning that infection occurs mainly from environmental sources. However, molecular epidemiology studies suggested that infections occur predominantly in a cow-to-cow fashion. *S. agalactiae* has been considered an extremely contagious mastitis pathogen for several years, and an important human pathogen. It was reported that some clonal complexes of *S. agalactiae* were shared between cows and farm personnel, indicating the zoonotic potential of this species (Cobo-Angel et al., 2019). These pathogens are also producers of biofilms (Gomes et al., 2016; Reinoso, 2017; Alves-Barroco et al., 2019; Horiuk et al., 2019), and this virulence characteristic has been associated with persistent infections and development of antibiotic resistance (Olson et al., 2002; Ruppen et al., 2017).

## Increased Tolerance and Resistance Against Antimicrobial Agents

Overall, upon biofilm formation, there is a delayed internalization of the antimicrobial through the biofilm matrix, as the primary physical and/or chemical diffusion barrier prevents the entrance of polar and charged antibiotics (Mah and O’Toole, 2001; Patel, 2005; Høiby et al., 2010). Additionally, the heterogeneous growth of the biofilm cells and activation of the stress response genes contribute to the resistance phenotype (Bjarnsholt et al., 2013a, b; Macià et al., 2014). Besides, studies show that a biofilm-specific phenotype is induced in a subpopulation, and it results in the differential expression of active mechanisms to combat the effects of antimicrobial agents (Konto-Ghiorghi et al., 2009; Genteluci et al., 2015).

The role of biofilm in evading conventional antimicrobials for the treatment against streptococcal infections is not yet entirely understood, although several studies have shown that streptococcal biofilms survive after treatments with high concentrations of antibiotics (Ogawa et al., 2011; Horiuk et al., 2019). Overall, this resistance mechanism is the consequence of the multicellular and matrix nature of biofilms, which leads to the antibiotic resistance of biofilm communities, along with the known conventional resistance mechanisms, such as, efflux pumps, modifications of the antimicrobial target, and enzyme inactivation (Rosini and Margarit, 2015; Young et al., 2016). The main mechanisms of resistance in pyogenic streptococci are revised in Alves-Barroco et al., 2020. Several *in vitro* studies demonstrated that when enclosed and protected by a biofilm, bacteria are 10–1000 times more resistant to antimicrobial drugs when compared to the planktonic counterpart (Olson et al., 2002; Baldassarri et al., 2006; Shen et al., 2013; Chadha, 2014; Wu et al., 2015; Boonyayatra and Pata, 2016; Singh et al., 2017). Macià et al. (2014) proposed a method for selecting antibiotics against biofilm bacteria which can also be used for developing new antimicrobial agents. This strategy is based on assessing six pharmacodynamic parameters:

i minimal inhibitory concentration (MIC), can be defined as the lowest concentration of an antimicrobial that inhibits the growth of planktonic cells;
ii minimal biofilm inhibitory concentrations (MBIC), which is the lowest concentration of an antimicrobial that results in a difference of 1 log in growth after six h of incubation;
iii minimal bactericidal concentration (MBC) is the lowest concentration of an antimicrobial that killing 99.9% of the colony-forming units (CFUs);
iv biofilm bactericidal concentration (BBC) is the lowest concentration of an antimicrobial producing a 99.9% reduction of the CFUs from a biofilm culture when compared to the control;
v minimum biofilm eradication concentration (MBEC) is the lowest concentration of an antimicrobial that inhibits visible after to collect biofilm cells;
vi biofilm-prevention concentration (BPC) is the similar as the MBIC, but bacterial inoculation and antimicrobial exposure co-occur (Macià et al., 2014).

To the best of our knowledge, the information available regarding the MBEC of pyogenic streptococcal is scarce. However, some studies indicate that biofilms produced by pyogenic streptococci are resistant to various antibiotics (Conley et al., 2003; Baldassarri et al., 2006; Pichichero and Casey, 2007; Horiuk et al., 2019), suggesting that most of the antibiotics evaluated would be ineffective as antimicrobial agents.

The β-lactams (mainly penicillin) have been universally accepted as the antibiotics of choice for pyogenic streptococci infections; however, therapeutic failures have been reported and attributable to different causes, including biofilm formation (Pichichero and Casey, 2007; Bonofiglio et al., 2018; Moroi et al., 2019). Conley and coworkers reported penicillin treatment failure in 32% of case-patients with *S. pyogenes* infection. The authors first reported *in vitro S. pyogenes* insensitivity to penicillin by MBEC assay (Conley et al., 2003). Torretta et al. (2012) reported pharyngitis treatment failure 37% of children to have biofilm producer *S. pyogenes* and with increased MBEC.

Macrolides are considered antibiotics of choice in human therapy for the treatment of pneumonia, sinusitis, and otitis in cases where patients are allergic to β-lactams (Kanoh and Rubin, 2010), and lincosamides are used as alternative to penicillin G against anaerobic bacteria and streptococci strains (Greenwood and Irving, 2012). However, therapeutic failures of macrolide have been suggested as a consequence of the formation of biofilms by *S. pyogenes*, resulting in clonal spread (Baldassarri et al., 2006). Some antibiotics, such as fluoroquinolones, and aminoglycosides, are not active in anaerobic conditions, affecting only the outer part of the biofilm (Borriello et al., 2004).

Ruppen and coworker compared MICs and MBECs using penicillin, gentamicin, and a combination of both among *S. agalactiae* biofilm-forming strains. The results showed reduced susceptibility to penicillin, and that the concentration of gentamicin against *S. agalactiae* biofilm cannot be achieved in bone with systemic administration, only administered locally (Ruppen et al., 2017).

Horiuk and coworkers analyzed resistance to antimicrobials among bovine mastitis pathogens biofilm producers. The results showed resistance to penicillin, aminoglycosides, and macrolides, emphasizing the necessity of alternative approaches and develop new antibiotics to effectively treat bovine mastitis (Horiuk et al., 2019). In addition, the indiscriminate administration of antibiotics in dairy cows various for the promotion of growth and treatment provides substantial residues of antibiotics that are released through milk of dairy and significant effects on human health (Sachi et al., 2019).

## Horizontal Gene Transfer is Increased in Biofilms

Biofilms increase HGT rates among community cells, by 16,000 times, due to high cell density and/or accumulation of genetic elements, either by transformation, conjugation or transduction, (Savage et al., 2013; Kragh et al., 2016; Emamalipour et al., 2020). Besides the genes involved in mobility, regulation or maintenance, mobile genetic elements (MGEs) provide the conditions for the uptake of antibiotic-resistant genes and virulence factors (Haenni et al., 2010). Thus, HGT has been found to be a major mechanism in generating gene diversity through intragenic and intergenic recombination, which can modulate host-pathogen interactions and extending the host range. Such gene transfer events frequently occur within the pyogenic group, particularly in *S. pyogenes, S. agalactiae*, S*treptococcus canis*, SDSD, SDSE, S*treptococcus equi* subsp. *equi*, S*treptococcus equi* subsp. *zooepidemicus*, and *S. uberis* (Baiano and Barnes, 2009; Haenni et al., 2010; Rato et al., 2010; Wong and Yuen, 2012; Richards et al., 2014; Rohde and Cleary, 2016).

In *S. pyogenes* the lateral exchange of virulence genes, mediated by bacteriophage infection, is a very important factor in the diversification of the species (McShan and Nguyen, 2016). Bacteriophages may convey genes that are advantageous to the hosts, thus fostering their own dissemination (Von Wintersdorff et al., 2016). An extreme case is presented by *S. pyogenes* in which almost all major gaps in the alignment of different M serotypes could be traced to prophage integration events (Canchaya et al., 2004). Marks and coworkers demonstrated that the biofilm microenvironment of *S. pyogenes* populations results in the induction of competence genes; therefore, it is more conducive to HGT. This study shows for the first time that *S. pyogenes* can be naturally transformed in the presence of exogenous DNA when grown as biofilms both *in vitro* and *in vivo* (Marks et al., 2014a).

Horizontal gene transfer also appears to play a role in the evolution and population structure of SDSE. Studies have revealed that HGT and recombination occur between *S. pyogenes* and SDSE, indeed, the two share several virulence factors and are coexist in the human host (McNeilly and McMillan, 2014).

We reported in previous studies for the first time the ability of milk udder SDSD isolates containing phage-encoded *S. pyogenes* genes to adhere and internalize primary human keratinocytes (Roma-Rodrigues et al., 2016) and niches of colonization/infection of *S. pyogenes* from the respiratory track: Detroit 562, a cell line derived from the metastatic site of pharynx carcinoma, primary Bronchial/Tracheal Epithelial Cells (BTEC), and A549, a cell line derived from a human adenocarcinoma of the alveolar basal epithelial cells (Alves-Barroco et al., 2018). Several genes are responsible for the increased virulence of these subspecies, providing the dissemination to a different host and propagating the infection persistent (Rato et al., 2011; Alves-Barroco et al., 2018). This subspecies has been considered by some authors as an emerging zoonotic pathogen (Chennapragada et al., 2018). Moreover, their biofilm-producing ability (Alves-Barroco et al., 2019) can make them highly difficult to eradicate.

The successful treatment of biofilm-associated streptococcal infections is troubled due to high antibiotic resistance. Conventional antibacterial therapy is unable to fully eradicate biofilms cells. Therefore, to overcome the resistance of biofilm, alternative strategies and antibiofilm agents have been studied.

## Emergent Alternatives to Fight Biofilm Infection

Despite the increasing recognition of the impact of pyogenic infections, namely when adopting the biofilm phenotype, recurrent infections, uncontrolled contagion, therapeutic failure and morbidity, motivated scientists to find new solutions for this old problem. Some alternatives of antibiotics to combat biofilm-based infections are reviewed in Alves-Barroco et al. (2020). In the next paragraphs we want to give special attention to the protective effect of probiotic bacteria against pyogenic bacteria.

The use of live organisms as therapeutic agents has been accepted for many years, with more emphasis on the gut and oral cavity pathologies (Hoare et al., 2017). Since the last decade, studies have emerged describing how streptococcal infection can be overwhelmed by non-pathogenic bacteria, namely the resident bacterial species that form the microbiome. In 2013, the *in vitro* studies of Fiedler et al. (2013) showed that in direct contact experiments, *S. salivarius* and *S. oralis* in the planktonic phenotype eliminated the growth of *S. pyogenes*, and reduced its cell adhesion to eukaryotic laryngeal epithelial cell line (Hep2). In fact, the authors suggest that it is the structure of the *S. salivarius* biofilm covering the eukaryotic cells that protects them from the pathogen through steric hindrance. Recently, *Lactobacillus plantarum*, *Lactobacillus acidophilus*, and *S. salivarius* were tested for inhibition toward *S. pyogenes* cultures in deferred antagonism agar assays. The results show that these strains have anti-biofilm effects, especially *S. salivarius* (Humphreys and McBain, 2019). The antagonistic effect was not dependent on cell–cell contact since the pathogen and the probiotic were not in direct contact, but separated by a semi-permeable membrane that allowed diffusion of bacteriocins as salivaricin A2 (SalA2) and salivaricin B (SboB) in the case of *S. salivarius* K12 (Hyink et al., 2007), acidocins (Acedo et al., 2015) and plantaricins (Lages et al., 2015), in the case of of *L. acidophilus* and *L. plantarum*, respectively (in all cases, the acidification of the media was not observed). Effectiveness of the probiotic *Streptococcus salivarius* K12 for the treatment and/or prevention of sore throat was recently reviewed due to the large number of studies reporting the use of this agent in the treatment/prevention of upper respiratory tract infections (Wilcox et al., 2019). Despite describing *S. salivarius* K12 as being safe and well tolerated, the main conclusion of this systematic review of the literature is that this organism might have a prophylactic/therapeutic effect on sore throat in adults and children (namely derived from streptococcal infections), but more reliable and unbiased trials are required to establish this relation and boot this probiotic for regular use.

New targets for the development of new drugs are also gaining scientists attention. The LytR-A-Psr (LCP) family of proteins, are responsible for the insertion of WTAs and other anionic polymers into the peptidoglycan and have been associated with host cell adhesion and biofilm development (Chatfield et al., 2005). The *brpA*-like gene encoding the biofilm regulatory proteins A – homologous of the LytR protein – is a member of the LCP family (Shemesh et al., 2007). The *brpA* gene encodes a membrane associated protein (BrpA – biofilm regulator protein A) that has been implicated in biofilm formation, autolysis, and cell division in *S. mutans*. The deficiency of BrpA decreases the ability of the response to oxidative stress, cell envelope stress, and pH alteration. *In vitro*, the BrpA-deficient mutant can adhere to the surface, but its ability to form mature biofilm decreases considerably (Bitoun et al., 2012, 2014). Recently, we have reported that *brpA*-like gene is harbored by SDSD biofilm-producing strains, and its expression levels are associated with the biofilm-forming ability (Alves-Barroco et al., 2019). Structural and functional data on these and similar proteins will be an asset for the development of new antimicrobials with high specificity toward pyogenic infection, decreasing biofilm formation, affecting bacteria viability and decreasing antibiotic tolerance.

In addition, many pyogenic streptoccocci adhesins have been studied as antigens to vaccine potential, the most advanced candidates, having entered clinical trials, are based on the M and M-like proteins, while fibronectin-binding proteins and components of the pilus are in pre-clinical development (Frost et al., 2018; Raynes et al., 2018).

## Conclusion

Biofilms play an essential role in streptococci pathogenesis, contributing to therapy failure and promoting persistent infections. Although the influence of variations in environmental conditions has been extensively studied on planktonic phenotype, further information about how these variations modulate streptococci biofilm development is needed. It is known that multiple virulence factors modulate biofilm formation by pyogenic streptococci; however, there are missing pieces about biofilm formation and its impact on streptococcal disease. Details on mechanical, chemical, physical, genetic, and structural properties of pyogenic streptococci biofilm will allow envisioning new therapeutic strategies to prevent biofilms formation or to eradicate mature biofilm.

## Author Contributions

AF and TS-S conceived the idea. CA-B and JP-F wrote the draft. Revision by AF and TS-S. All authors contributed to the article and approved the submitted version.

## Conflict of Interest

The authors declare that the research was conducted in the absence of any commercial or financial relationships that could be construed as a potential conflict of interest.
